# Notes on *Glaucocharis* (Lepidoptera, Crambidae) from China, with descriptions of two new species

**DOI:** 10.3897/zookeys.807.29237

**Published:** 2018-12-17

**Authors:** Wei-Chun Li

**Affiliations:** 1 College of Agronomy, Jiangxi Agricultural University, Nanchang, 330045, China Jiangxi Agricultural University Nanchang China

**Keywords:** Pyraloidea, Crambinae, taxonomy, geographical distribution, China

## Abstract

Two new species belonging to the genus *Glaucocharis* Meyrick, 1938 are described from southwest China: *Glaucocharissperlingi***sp. n.** and *G.nussi***sp. n.** The female of *G.castaneus* Song & Chen, 2002 is described for the first time. The geographical distribution of the genus in China is analysed. The precipitation of the warmest quarter is revealed to be the strongest predictor affecting the present distribution pattern of the genus. A map showing the distribution of the known Chinese localities of *Glaucocharis* is provided.

## Introduction

The genus *Glaucocharis*, one of the most species-rich genera of the subfamily Crambinae (Lepidoptera, Crambidae), was established by Meyrick (1938) with *Glaucocharisstella* Meyrick, 1938 as the type species. To date, the genus has 153 described species worldwide (Nuss et al. 2018). The main taxonomic contributions concerned the faunas of the Palaearctic and Oriental Regions (Bleszynski 1965; Gaskin 1974b; Wang and Song 1983; Ganev 1987; Wang et al. 1988; Song 1993; Chen et al. 2001, 2002, 2003; Sasaki 2007; Li and Li 2012; Park et al. 2018), followed by the Australian Region (Gaskin 1971, 1974a, 1974b, 1985) and the Ethiopian Region (Bleszynski 1966, 1970; Bassi and Mey in Mey 2011). In China, the first specimen of *Glaucocharis* was recorded as early as 1932 from Tianmushan, Zhejiang Province (Bleszynski 1965), and a total of sixty species have been recorded in the country prior to this study (Li and Li 2012). Among them, forty-nine species have China as the type locality (Suppl. material 1: Table S1). To date, all known localities of Chinese *Glaucocharis* clearly indicate a mostly eastern distribution, but this geographical pattern was never previously mentioned.

Morphologically, the members of the genus can be recognized by characters of the forewing: the apex usually with an apical mark, the termen below the apex with an indentation reaching the tip of M_1,_ and well-developed marginal spots. In the wing pattern, *Glaucocharis* is similar to *Roxita* Bleszynski, but can be distinguished by the forewing with a well-developed M_1_ and the valva without a ventral fold in the male genitalia; in *Roxita*, M_1_ in the forewing is absent and the ventral fold of the valva is often present (Li and Li 2012). Several species groups have been proposed based on external characters and genitalic structures within *Glaucocharis* by Gaskin (1985) and Wang et al. (1988). However, it is relatively difficult to assign some ambiguous members to any proposed group on morphological characters alone. There is a need for a more insightful classification of species groups in this large genus based on an integrative approach using molecular data and morphological characters.

In the present paper, two species of *Glaucocharis* are described from the Mabian Dafengding National Nature Reserve, southwest of China. The geographical pattern of distribution presented by the genus in China is also commented upon.

## Material and methods

All specimens were collected at night with a mercury-vapour lamp. The specimens were hand-collected alive and killed by ammonium hydroxide just prior to mounting and spreading as shown in Landry and Landry (1994). The terminology for morphological structures follows Bleszynski (1965) and Gaskin (1985). Photographs of adults were taken with a Zeiss AxioCam Icc 5 digital camera attached to a Zeiss SteREO Discovery V12 microscope. Illustrations of the genitalia were prepared with a DV320 OPTPro2010-Chs digital camera attached to an Optec BK-DM320 microscope. All specimens examined are deposited in the Insect Museum, Jiangxi Agricultural University, Nanchang, China (JXAUM).

The distribution of *Glaucocharis* was analysed using MaxEnt (Phillips et al. 2006) and was based on distributional data extracted from previous studies (Bleszynski 1965; Wang and Song 1983; Wang et al. 1988; Song 1993; Chen et al. 2001, 2002, 2003; Li and Li 2012), the specimens examined in this study (Suppl. material 1: Table S1), and twenty environmental variables (Suppl. material 1: Table S2). Geographic coordinates were taken from Wu et al. (1997) and converted into decimal coordinates (Suppl. material 1: Table S1). MaxEnt was set with 10 000 as the maximum number of background points. The model’s goodness-of-fit and the relative importance of each of the variables were evaluated by area under the receiving operator curve and the jackknife procedure, respectively. Climatic data were retrieved from the WorldClim database (http://www.worldclim.org) at a resolution of 10 arc-min (Hijmans et al. 2004). The cartographic illustration was created using *dismo* R package (Hijmans and Elith 2017).

## Taxonomic account

### Glaucocharis sperlingi sp. n.

Taxon classificationAnimaliaLepidopteraCrambidae

http://zoobank.org/AF440590-1E0A-4AD5-B7ED-AA21C1C75C58

Figs 1 , 2 , 7

#### Type material.

*Holotype* ♂: CHINA: Mabian Dafengding National Nature Reserve, Mabian (28°51'N, 103°31'E), Sichuan Province, 1100 m, 12.viii.2012, coll. Wei-Chun Li, prep. gen, WD16102 (JXAUM).

*Paratype*, 1 ♂, with same locality as holotype and collected on 10.viii.2012 (JXAUM).

#### Diagnosis.

This new species is similar to *Glaucochariselectra* (Bleszynski) by having slender uncus and gnathos, and thin and long valva in the male genitalia. It can be distinguished by the basal process of the costa of the valva with two projections, the juxta ending with three spine-like projections, and the phallus with a line of tiny spine-like cornuti in the male genitalia (Fig. 7). In *G.electra*, the costa of the valva only has a single projection, the juxta is concave distally, and the phallus has only one cornutus (Bleszynski 1965: pl. 32 fig. 4).

#### Description.

**Figures 1–6.**
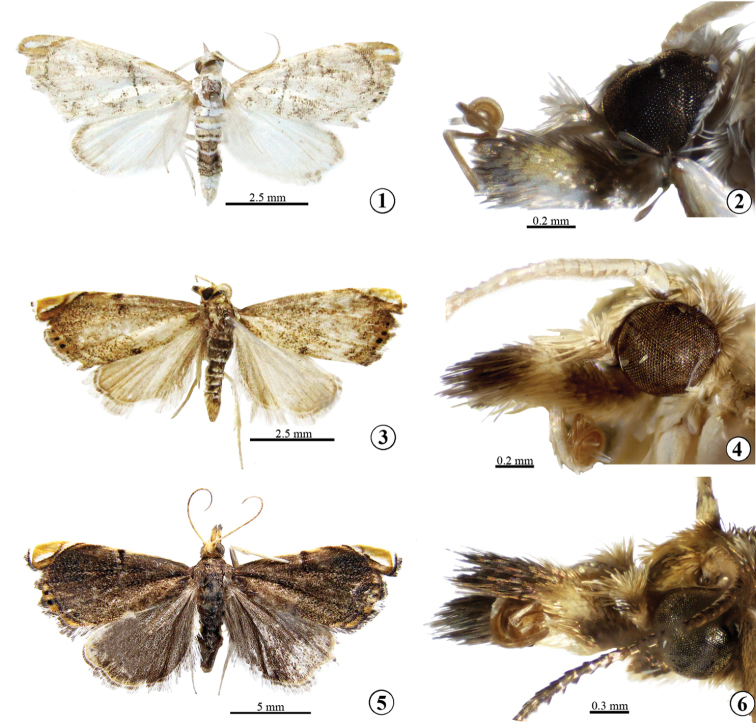
*Glaucocharis* spp. **1, 3, 5** Adult in dorsal view **2, 4, 6** Head in lateral view **1, 2***G.sperlingi* sp. n., male, holotype **3, 4***G.nussi* sp. n., male, holotype **5, 6***G.castaneus* Song & Chen, female.

*Male adult* (Figs 1, 2): Forewing length 5.5–6.0 mm. Frons and vertex pale brown. Labial palpus pale yellow on outer side except for brown base and tip, white ventrally. Maxillary palpus pale brown, white distally. Antenna pale brown and yellowish white in alternance on dorsal surface, pale yellow ventrally. Tegula and thorax white mixed with pale brown. Forewing white, sparsely covered with pale brown scales; antemedian line pale brown, straight except curved inward near costa; reniform stigma pale brown, small and ovate; postmedian line pale brown, arched outward; apex pale yellow, with white apical stripe; termen pale brown, with two black marginal spots; fringe pale brown mixed with white. Hindwing white, covered with pale brown scales along apex; fringe concolourous with forewing. Abdomen brown and white in alternance on dorsal surface. Legs white.

**Figures 7–9.**
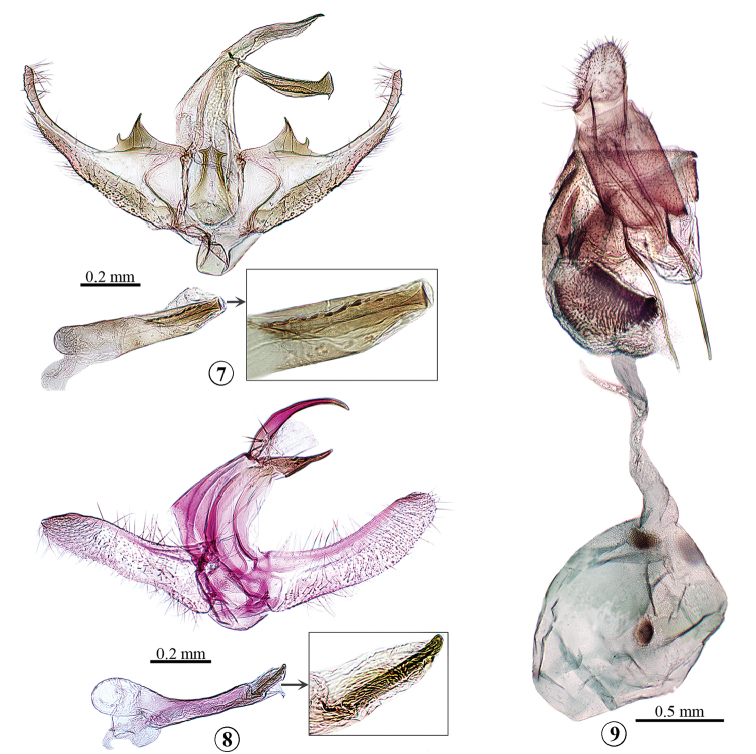
Genitalia of *Glaucocharis* species. **7***G.sperlingi* sp. n., male, holotype **8***G.nussi* sp. n., male, holotype **9***G.castaneus* Song & Chen, female.

*Male genitalia* (Fig. 7): Uncus slightly concave at two-thirds, tapering to pointed apex. Gnathos curved upward slightly, apex with triangular projection and small spine on dorsal and ventral margin, respectively. Tegumen approximately as long as gnathos. Valva broad basally, narrowed towards blunt apex; ventral margin indented at about three-fourths; costa with adjacent triangular and spine-like projections at base. Saccus well-developed, gently narrowed towards distal tip. Juxta anteriorly convex, slightly broadened in basal one-third, then narrowed towards tip, ending with three spine-like projections. Phallus slightly shorter than valva; tiny cornuti spine-like, placed in one line.

Female unknown.

#### Distribution.

China (Sichuan).

#### Natural history.

**Figure 10.**
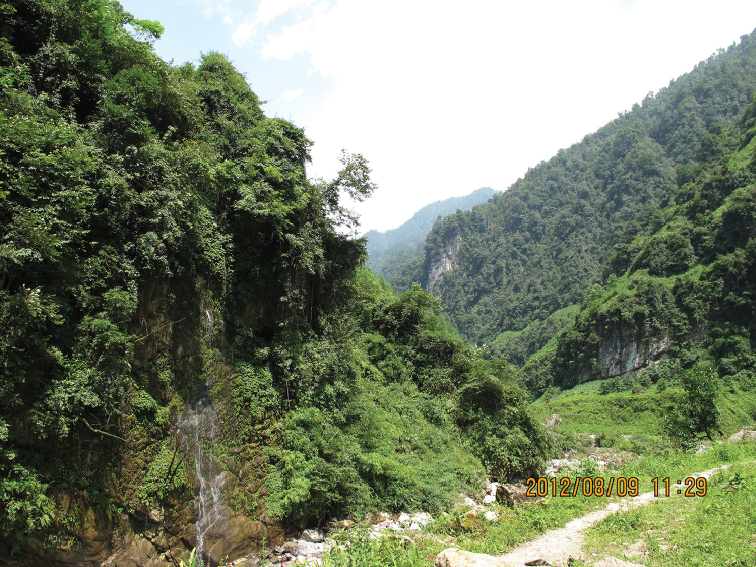
Collecting locality of the specimens treated here (Mabian Dafengding National Nature Reserve, Sichuan Province, China).

Unknown except that the moths are in flight in early August and come to light. The habitat in which this species has been collected is located at 1100 m altitude, at the foot of the mountain. Most parts of the mountain are covered with trees belonging to families Lauraceae and Fagaceae (Fig. 10).

#### Etymology.

In honour of Professor Felix Sperling of the University of Alberta, Canada, who contributed profoundly to systematic research in entomology, and who maintains long-standing achievements as curator of the E. H. Strickland Entomological Museum (http://www.entomology.museums.ualberta.ca).

### Glaucocharis nussi sp. n.

Taxon classificationAnimaliaLepidopteraCrambidae

http://zoobank.org/0DAE5EE5-442C-4B77-B86A-486B68E7F89A

Figs 3 , 4 , 8

#### Type material.

*Holotype* ♂: CHINA: Mabian Dafengding National Nature Reserve, Mabian (28°51'N, 103°31'E), Sichuan Province, 1100 m, 11.viii.2012, coll. Wei-Chun Li (JXAUM).

*Paratype*, 1 ♂, with same locality as holotype and collected on 10.viii.2012, prep. gen. WD16100 (JXAUM).

#### Diagnosis.

This species can be distinguished from its congeners by the unique characters in the male genitalia. The costal projection is absent and the phallus has a single strong spine-like cornutus (Fig. 8).

#### Description.

*Male adult* (Figs 3, 4): Forewing length 5.5–6.0 mm. Frons and vertex pale brown mixed with yellowish white. Labial palpus basal half and distal one-fourth blackish brown on outer side, otherwise yellowish white. Maxillary palpus pale brown, yellowish white distally. Antenna yellowish white. Tegula and thorax pale brown. Forewing covered with pale brown scales; costa and dorsum with blackish spot near middle; antemedian line unrecognized; reniform stigma blackish brown, small and round; postmedian line brown, arched outward; apex orange, with white apical stripe; termen orange mixed with pale brown, with four black marginal spots; fringe pale brown. Hindwing pale brown; fringe white mixed with grey. Abdomen blackish brown and white in alternance on dorsal surface. Legs pale brown.

*Male genitalia* (Fig. 8): Uncus curved downward, tapering to pointed apex. Gnathos nearly narrowly triangular, pointed distally. Tegumen approximately twice as long as gnathos. Valva broadened slightly at base, apex round; costa concave near middle. Saccus short and broad, convex distally. Juxta crescent-shaped. Phallus nearly as long as valva, basal one-third conspicuously thicker than distal two-thirds; single cornutus well-developed and spine-like.

Female unknown.

#### Distribution.

China (Sichuan).

#### Natural history.

See above under this heading for *Glaucocharissperlingi* sp. nov.

#### Etymology.

In honour of Dr Matthias Nuss, who contributed profoundly to systematic research on pyraloid moths, and who maintains and expands the most important tool for taxonomic information on the world pyraloid species: GlobIZ (www.pyraloidea.org).

#### Remarks.

The generic assignment of *G.nussi* is primarily based on the external characters. However, its male genitalia are atypical for *Glaucocharis*. Characters of both sexes and molecular data would have to be analysed phylogenetically to provide a more insightful hypothesis concerning its classification.

### Glaucocharis castaneus

Taxon classificationAnimaliaLepidopteraCrambidae

Song & Chen, 2002

Figs 5 , 6 , 9

Glaucocharis castaneus Song & Chen, in Chen et al. 2002: 1, figs 1–3. Type locality: Maoershan, Guangxi Province, China.

#### Specimens examined.

23 ♂♂, 12 ♀♀: CHINA: Mabian Dafengding National Nature Reserve, Mabian (28°51'N, 103°31'E), Sichuan Province, 1100 m, 9−10.viii.2012, coll. Wei-Chun Li (JXAUM).

#### Description.

*Female adult* (Figs 5, 6): Forewing length 6.5−8.0 mm. Frons and vertex pale yellow. Labial palpus blackish-brown except second segment pale yellow. Maxillary palpus pale brown to blackish brown, pale yellow distally. Antenna blackish brown and pale yellow in alternance on dorsal surface, pale yellow ventrally. Tegula and thorax blackish brown. Forewing densely covered with blackish brown scales; antemedian line black, dorsal two-thirds inconspicuous; reniform stigma unrecognized; postmedian line blackish brown, arched outward; apex orange, with white apical stripe; termen orange mixed with pale brown, with two black marginal spots; fringe blackish brown. Hindwing blackish brown; fringe pale brown except blackish brown subbasally. Abdomen blackish brown on dorsal surface. Legs pale brown.

*Female genitalia* (Fig. 9): Papillae anales ovate, about one-third length of posterior apophyses. Tergite eight about two-thirds as long as anterior apophyses. Lamella postvaginalis roughly U-shaped. Antrum swollen and densely covered with small spines. Ductus bursae long and thin. Ductus seminalis arising from approximately posterior one-third of ductus bursae. Corpus bursae rounded; signa double and ovate.

#### Distribution.

China (Guangxi, Sichuan).

#### Remarks.

The female of *G.castaneus* is described for the first time herein. The male of this species was described and figured adequately by Chen et al. (2002).

## The geographical distribution of *Glaucocharis* in China

The geographical distribution of Chinese *Glaucocharis* was analysed using MaxEnt based on the known localities (Suppl. material 1: Table S1) and twenty environmental variables (Suppl. material 1: Table S2). The results clearly indicate that the precipitation of the warmest quarter (Bio18) was the strongest predictor of the geographical distribution of the genus in China, and the mean diurnal range (Bio2, mean of monthly maximum and minimum temperatures) and the minimum temperature of the coldest month (Bio6) were revealed to be the second and third most important factors respectively in the environmental variables (Suppl. material 1: Table S2).

**Figure 11.**
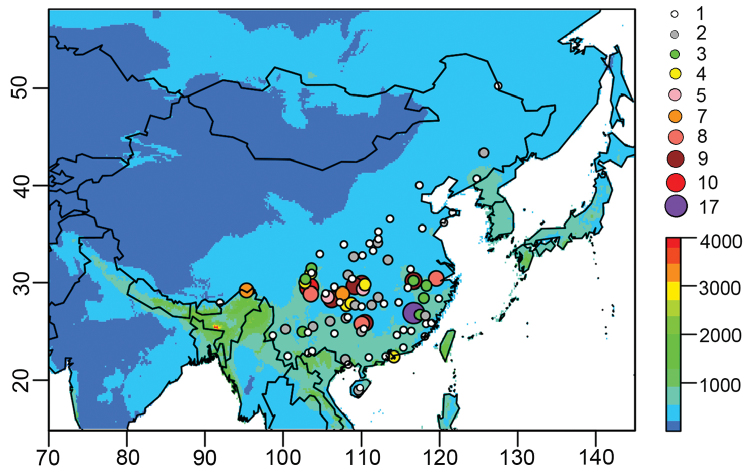
Geographical distribution of *Glaucocharis* in China and precipitation of the warmest quarter (Bio18). Circles indicate surveyed sites and numbers of species per site. Rainbow bar: precipitation (mm).

At present, all *Glaucocharis* species in China occur in humid–semi-humid areas (pale blue to green), which can be separated from arid–semi-arid areas (dark blue) in western China by using the climate data Bio18 (Fig. 11). Furthermore, based on all *Glaucocharis* species catalogued in China (Suppl. material 1: Table S1), most members of the genus occur south of 32°N (southern China) where the minimum temperature of the coldest month is above 0 °C. The precipitation and temperature have higher explanatory power for the occurrence of the genus in China in accordance with the analysis of MaxEnt. The available data suggest that precipitation limits the dispersal of known species. Meanwhile, temperature could have a significant influence on the exceptionally high species diversity of the genus in southern China. However, the species diversity pattern of *Glaucocharis* detected here does not precisely reflect the latitudinal gradient inasmuch as the unique species diversity between 25°N and 32°N is much higher than in the other areas, and many distribution gaps are found between the known localities (Fig. 11). In further research, it would be essential to explore more precisely the biotic and abiotic requirements for individual *Glaucocharis* species as well as to describe the largely unstudied diversity of the genus in eastern China.

## Supplementary Material

XML Treatment for Glaucocharis sperlingi

XML Treatment for Glaucocharis nussi

XML Treatment for Glaucocharis castaneus

## Supplementary materials

Supplementary material 1 Supplementary tables Data type: species data Explanation note: **Table S1.** Overview of Chinese localities where Glaucocharis species have been collected (administrative divisions are given in bold) with geographical coordinates in the decimal system; **Table S2.** Environmental variables used in the study and their contribution percentage and permutation importance. This is an open access article distributed under the terms of the Creative Commons Attribution License (CC BY 4.0), which permits unrestricted use, distribution, and reproduction in any medium, provided the original author and source are credited.Wei-Chun Li
