# Supplementary material for: Notes on Glaucocharis (Lepidoptera, Crambidae) from China, with descriptions of two new species
Source: Zookeys. 2018 Dec 17;(807):149–58. doi: 10.3897/zookeys.807.29237 (PMC6305352; doi:10.3897/zookeys.807.29237)
Supplement: Supplementary material 1 — Supplementary tables [file zookeys-807-149-s001.docx]

**Notes on *Glaucocharis* (Lepidoptera, Crambidae) from China, with descriptions of two new species**

**Supporting Information**

**Table S1.** Overview of Chinese localities where *Glaucocharis* species have been collected (administrative divisions are given in bold) with geographical coordinates in the decimal system

| **Taxa** | **Collection locality** | **Eastern Longitude** | **Northern Latitude** |
| --- | --- | --- | --- |
| *G. alatella* Wang & Sung* | **Sichuan**, Emeishan | 103.3117 | 29.5333 |
| *G. albilinealis* (Hampson)**^#^** | **Sichuan** | - | - |
| *G. apicudentella* Song & Chen* | **Guangxi**, Maoershan | 110.4167 | 25.8833 |
| *G. assamensis* Gaskin | **Guizhou**, Mayanghe, 700 m | 109.2000 | 27.7167 |
|  | **Sichuan**, Emeishan | 103.3117 | 29.5333 |
| *G. baculella* Wang & Sung* | **Fujian**, Wuyishan | 116.7000 | 26.9000 |
|  | **Guangxi**, Longsheng | 110.0333 | 25.7833 |
|  | **Guizhou**, Leishan, 900 m | 108.0500 | 26.3667 |
|  | Leigongshan, 1600 m | 108.2833 | 26.4667 |
| *G.* *biconvexa* Li & Li* | **Anhui**, Huangshan | 118.3000 | 29.7167 |
|  | **Fujian**, Daiyunshan, 850 m | 118.2500 | 25.7833 |
|  | Shanghang, 600 m | 116.4000 | 25.0500 |
|  | Wuyishan, 600 m | 116.7000 | 26.9000 |
|  | **Guangxi**, Leye | 106.5667 | 24.7833 |
|  | Shangsi | 107.9667 | 22.1500 |
|  | Yongfu, 160 m | 109.9833 | 24.9833 |
|  | **Guizhou**, Chishui, 240 m | 105.7000 | 28.5667 |
|  | Mayanghe, 430 m | 109.2000 | 27.7167 |
|  | Xishui, 500 m | 106.2000 | 28.3167 |
|  | **Hong Kong**, New Territories, 130–700 m | 114.1667 | 22.4167 |
|  | **Hunan**, Taojiang | 112.1333 | 28.5333 |
|  | Xinhua | 111.3000 | 27.7333 |
|  | **Jiangxi**, Guanshan | 114.7833 | 28.0050 |
|  | Saibaishan, 600 m | 115.4100 | 25.1500 |
|  | **Shaanxi**, Baihe | 110.1167 | 32.8167 |
| *G. bifidella* Wang & Sung* | **Sichuan**, Dukou | 106.0000 | 26.0500 |
| *G. brevis* Li & Li* | **Guizhou**, Daozhen, 1450 m | 107.6000 | 28.8833 |
|  | Suiyang, 840–1500 m | 107.2500 | 28.0167 |
| *G. castaneus* Song & Chen* | **Guangxi**, Longsheng | 110.0333 | 25.7833 |
|  | Maoershan | 110.4167 | 25.8833 |
|  | **Sichuan**, Mabian,1100 m | 103.5167 | 28.8500 |
|  | Wanxian | 108.3000 | 30.8000 |
|  | **Yunnan**, Kunming | 102.4500 | 24.9667 |
| *G. chelatella* Wang & Sung* | **Shaanxi**, Hualongshan, 800 m | 109.0167 | 32.6833 |
|  | **Yunnan**, Jinping | 103.2000 | 22.7833 |
| *G. copernici* (Bleszynski)* | **Guizhou**, Daozhen, 1300 m | 107.6000 | 28.8833 |
|  | **Tibet**, Mêdog, 1100 m | 95.3000 | 29.2167 |
|  | **Zhejiang**, Tianmushan | 119.5667 | 30.4333 |
| *G. electra* (Bleszynski)* | **Fujian**, Wuyishan, 1100 m | 116.7000 | 26.9000 |
|  | **Guangxi**, Longsheng | 110.0333 | 25.7833 |
|  | **Guizhou**, Daozhen, 1350–1370 m | 107.6000 | 28.8833 |
|  | **Hainan**, Diaoluoshan, 940 m | 109.8667 | 18.7833 |
|  | **Henan**, Guanshan, 550 m | 114.7833 | 28.0050 |
|  | Huaguoshan, 1100 m | 112.1667 | 34.5167 |
|  | Lushi, 1100 m | 111.0333 | 34.0500 |
|  | Tongbai, 300 m | 113.4000 | 32.3500 |
|  | Wangwushan, 800–1100 m | 112.1667 | 35.2667 |
|  | **Hubei**, Hefeng, 1260 m | 110.0333 | 29.8833 |
|  | Shennongjia, 1100–1400 m | 116. 6667 | 30.2000 |
|  | Wufeng, 1000 m | 116.6667 | 30.2000 |
|  | Xianfeng, 1280 m | 109.1333 | 29.6667 |
|  | **Hunan**, Sangzhi, Badagongshan, 1250 m | 110.1833 | 29.3833 |
|  | Zhangjiajie, 650 m | 110.4333 | 29.8167 |
|  | **Shandong**, Qingdao | 120.5500 | 36.2000 |
|  | Yantai | 121.6667 | 37.2000 |
|  | **Shaanxi**, Hualongshan, 800 m | 109.0167 | 32.6833 |
|  | **Sichuan**, Mabian, 900 m | 103.5167 | 28.8500 |
|  | Tianquan, 1300 m | 102.7667 | 30.0100 |
|  | **Tianjin**, Jixian, Baxianshan, 560 m | 117.4000 | 40.0333 |
|  | **Zhejiang**, Tianmushan, 350–800 m | 119.5667 | 30.4333 |
| *G. exsectella* (Chistoph) | **Fujian**, Wuyishan | 116.7000 | 26.9000 |
|  | **Heilongjiang**, Heihe, Kalunshan, 120 m | 127.4833 | 50.2333 |
|  | **Liaoning**, Kuandian | 124.7667 | 40.7167 |
|  | **Jilin**, Changbaishan | 125.6500 | 43.3500 |
| *G. flavifasciaria* Li & Li* | **Guizhou**, Xishui, 1200 m | 106.2000 | 28.3167 |
| *G. forcipella* Wang & Sung* | **Guangxi**, Maoershan | 110.4167 | 25.8833 |
|  | **Sichuan**, Qingchengshan | 103.5167 | 30.9667 |
| *G. furculella* Wang & Sung* | **Yunnan**, Qujing | 103.8000 | 25.5000 |
| *G. grandispinata* Li & Li* | **Yunnan**, Longling, Xiaoheishan, 2300 m | 98.6833 | 24.5833 |
|  | Kunming | 102.4500 | 24.9667 |
| *G. hastatella* Song & Chen* | **Fujian**, Wuyishan, 740–1100 m | 116.7000 | 26.9000 |
| *G. himalayana* Gaskin | **Zhejiang**, Tianmushan | 119.5667 | 30.4333 |
| *G. huanggangensis* Song & Chen* | **Fujian**, Wuyishan, 1800 m | 116.7000 | 26.9000 |
|  | **Guizhou**, Jiangkou, 1700 m | 108.0833 | 27.6833 |
|  | **Hubei**, Hefeng, 1260 m | 110.0333 | 29.8833 |
|  | Wufeng, 1000 m | 116.6667 | 30.2000 |
|  | Xianfeng, 1280 m | 109.1333 | 29.6667 |
| *G. incisella* (Bleszynski) | **Hainan**, Wuzhishan, 700 m | 109.6833 | 18.8833 |
|  | **Tibet**, Mêdog, 780–1100 m | 95.3000 | 29.2167 |
| *G. infundella* Wang & Sung* | **Sichuan**, Qingchengshan | 103.5167 | 30.9667 |
| *G. lasiotella* Song & Chen* | **Jiangxi**, Jiulianshan | 114.5500 | 24.6333 |
| *G. longqiensis* Song* | **Fujian**, Longqishan | 117.4667 | 26.7333 |
|  | Nanping, 850 m | 118.1667 | 26.6333 |
|  | Yongtai, Qingyunshan, 550 m | 118.9500 | 25.8667 |
|  | **Guangxi**, Maoershan | 110.4167 | 25.8833 |
|  | **Guizhou**, Chishui, 240–390 m | 105.7000 | 28.5667 |
|  | Xishui, 500 m | 106.2000 | 28.3167 |
|  | **Jiangxi**, Sanqingshan, 410–420 m | 117.9667 | 28.4500 |
|  | **Sichuan**, Emeishan | 103.3117 | 29.5333 |
| *G. lunatella* Wang & Sung* | **Sichuan**, Emeishan | 103.3117 | 29.5333 |
| *G. melistoma* (Meyrick)* | **Anhui**, Huangshan | 118.3000 | 29.7167 |
|  | **Fujian**, Wuyishan | 116.7000 | 26.9000 |
|  | **Gansu**, Wenxian, 860 m | 104.6833 | 32.9667 |
|  | **Guangxi**, Longsheng | 110.0333 | 25.7833 |
|  | Yongfu, 160 m | 109.9833 | 24.9833 |
|  | **Guizhou**, Daozhen, 1300–1350 m | 107.6000 | 28.8833 |
|  | Fanjingshan, 1300–2100 m | 108.6833 | 27.9167 |
|  | Jiangkou, 600–1700 m | 108.0833 | 27.6833 |
|  | **Hainan**, Diaoluoshan, 940 m | 109.8667 | 18.7833 |
|  | **Henan**, Songxian, Baiyunshan, 1400 m | 112.0833 | 34.1333 |
|  | **Hubei**, Hefeng, 1260 m | 110.0333 | 29.8833 |
|  | Wufeng, 1000 m | 116.6667 | 30.2000 |
|  | Xianfeng, 1280 m | 109.1333 | 29.6667 |
|  | **Hunan**, Sangzhi, Badagongshan, 1250 m | 110.1833 | 29.3833 |
|  | **Sichuan**, Baoxing | 102.8333 | 30.3667 |
|  | Guanxian | 103.6167 | 31.0167 |
|  | Mabian, 1500 m | 103.5167 | 28.8500 |
|  | Qingchengshan | 103.5167 | 30.9667 |
|  | Tianquan, 1300 m | 102.7667 | 30.0100 |
|  | **Yunnan**, Weishan, 2200 m | 100.3000 | 25.2333 |
|  | **Zhejiang**, Lin’an, 900 m | 118.9333 | 30.0667 |
|  | Lishui, Fengyangshan, 1470 m | 119.9000 | 28.4000 |
|  | Taishun, 680 m | 119.7000 | 27.5500 |
|  | Tianmushan, 350–1140 m | 119.5667 | 30.4333 |
| *G. melli* (Caradja)* | **Guangdong**, Sanjiang | 113.1000 | 22.4500 |
| *G. minutalis* (Hampson) | **Guangxi**, Maoershan | 110.4167 | 25.8833 |
|  | **Guizhou**, Xishui, 500 m | 106.2000 | 28.3167 |
| *G. moriokensis* (Okano) | **Anhui**, Jiuhuashan | 117.8000 | 30.3833 |
|  | **Fujian**, Longqishan | 117.4667 | 26.7333 |
|  | **Guizhou**, Chishui, 240 m | 105.7000 | 28.5667 |
|  | Xishui, 500 m | 106.2000 | 28.3167 |
|  | **Hebei**, Shexian, 700 m | 113. 6667 | 36.5667 |
|  | **Henan**, Xixia, 890 m | 111.4833 | 33.3000 |
|  | **Hubei**, Lichuan, 700 m | 108.9333 | 30.3000 |
|  | Xianfeng, 400 m | 109.1333 | 29.6667 |
|  | **Hunan**, Hupingshan, 504 m | 110.0167 | 27.5667 |
|  | Taojiang | 112.1333 | 28.5333 |
|  | Xiangtan | 112.8833 | 27.8667 |
|  | Xinhua | 111.3000 | 27.7333 |
|  | **Jiangxi**, Sanqingshan, 380–390 m | 117.9667 | 28.4500 |
|  | **Shandong**, Yimengshan | 117.7667 | 35.6167 |
|  | **Sichuan**, Luxian | 105.4333 | 28.9333 |
|  | Wanxian | 108.3000 | 30.8000 |
| *G. mutuurella* (Bleszynski) | **Fujian**, Wuyishan | 116.7000 | 26.9000 |
|  | **Hubei**, Hefeng, 1260 m | 110.0333 | 29.8833 |
|  | Shennongjia, 1100 m | 116. 6667 | 30.2000 |
|  | Wufeng, 1000 m | 116.6667 | 30.2000 |
|  | **Hunan**, Sangzhi, Badagongshan, 1250 m | 110.1833 | 29.3833 |
|  | **Zhejiang**, Tianmushan, 1140–1500 m | 119.5667 | 30.4333 |
| *G. nussi* Li, sp. nov.* | **Sichuan**, Mabian, 1100 m | 103.5167 | 28.8500 |
| *G. ochronella* Wang & Sung* | **Yunnan**, Pingbian | 103.6833 | 22.9667 |
| *G. octacornutella* Wang & Sung* | **Fujian**, Wuyishan | 116.7000 | 26.9000 |
|  | **Sichuan**, Dukou | 106.0000 | 26.0500 |
| *G. omeishani* (Bleszynski)* | **Fujian**, Wuyishan, 740 m | 116.7000 | 26.9000 |
|  | **Guizhou**, Daozhen, 1370 m | 107.6000 | 28.8833 |
|  | **Hubei**, Hefeng, 1260 m | 110.0333 | 29.8833 |
|  | **Sichuan**, Baoxing, 1100–1600 m | 102.8333 | 30.3667 |
|  | Mabian, 900–1500 m | 103.5167 | 28.8500 |
|  | Tianquan, 1300 m | 102.7667 | 30.0100 |
|  | Wolong, 1900 m | 103.6000 | 31.4833 |
| *G. palidella* Wang & Sung* | **Fujian**, Wuyishan, 740 m | 116.7000 | 26.9000 |
|  | **Guangxi**, Longsheng | 110.0333 | 25.7833 |
|  | **Guizhou**, Chishui, 240 m | 105.7000 | 28.5667 |
|  | Jiangkou, 600 m | 108.0833 | 27.6833 |
|  | Xishui, 550 m | 106.2000 | 28.3167 |
|  | **Hainan**, Diaoluoshan, 940 m | 109.8667 | 18.7833 |
| *G. parmulella* Wang & Sung* | **Guizhou**, Fanjingshan, 2100 m | 108.6833 | 27.9167 |
|  | **Hubei**, Xianfeng, 1280 m | 109.1333 | 29.6667 |
|  | Wufeng, 1100 m | 116.6667 | 30.2000 |
|  | **Shaanxi**, Huoditang | 108.4333 | 33. 4333 |
|  | **Sichuan**, Mabian | 103.5167 | 28.8500 |
| *G. parthenie* (Bleszynski)* | **Fujian**, Wuyishan | 116.7000 | 26.9000 |
| *G. paulispinata* Li & Li* | **Tibet**, Mêdog, 2380 m | 95.3000 | 29.2167 |
| *G. pilcheri* Gaskin | **Tibet**, Mêdog, 2380 m | 95.3000 | 29.2167 |
| *G. pomae* Wang & Sung* | **Tibet**, Bomi | 95.7500 | 29.8833 |
| *G. quadratella* Song & Chen* | **Guangxi**, Maoershan, 2141 m | 110.4167 | 25.8833 |
| *G. qujingella* Wang & Sung* | **Yunnan**, Qujing | 103.8000 | 25.5000 |
| *G. ramona* (Bleszynski)* | **Sichuan**, Emeishan | 103.3117 | 29.5333 |
|  | **Yunnan**, Lijiang | 100.2333 | 26.8667 |
| *G. rectifascialis* Gaskin | **Fujian**, Wuyishan | 116.7000 | 26.9000 |
|  | **Hubei**, Lichuan, 700 m | 108.9333 | 30.3000 |
|  | Xianfeng, 1280 m | 109.1333 | 29.6667 |
|  | **Hunan**, Sangzhi, Badagongshan, 1250 m | 110.1833 | 29.3833 |
|  | Zhangjiajie | 110.4333 | 29.8167 |
|  | **Guangxi**, Longsheng, 950 m | 110.0333 | 25.7833 |
|  | Jinxiu, 550 m | 110.1833 | 24.1333 |
|  | **Guizhou**, Leishan, 900 m | 108.0500 | 26.3667 |
|  | Fanjingshan, 1300 m | 108.6833 | 27.9167 |
|  | Xishui, 500–550 m | 106.2000 | 28.3167 |
|  | **Sichuan**, Emeishan | 103.3117 | 29.5333 |
|  | **Tibet**, Mêdog, 2380 m | 95.3000 | 29.2167 |
| *G. reniella* Wang & Sung* | **Fujian**, Wuyishan, 1400–1560 m | 116.7000 | 26.9000 |
|  | **Guangxi**, Guilin | 110.2902 | 25.2736 |
|  | **Guizhou**, Fanjingshan, 1300–2100 m | 108.6833 | 27.9167 |
|  | **Hainan**, Jianfengling, 940 m | 108.7833 | 18.7000 |
|  | **Henan**, Songxian, Baiyunshan, 1400 m | 112.0833 | 34.1333 |
|  | Neixiang, 1350 m | 111.0833 | 33.0333 |
|  | **Hubei**, Hefeng, 1260 m | 110.0333 | 29.8833 |
|  | Xianfeng, 1280 m | 109.1333 | 29.6667 |
|  | **Hunan**, Sangzhi, Badagongshan, 1250 m | 110.1833 | 29.3833 |
|  | **Jiangxi**, Sanqingshan, 1120 m | 117.9667 | 28.4500 |
|  | **Sichuan**, Wanzhou, Wangerbao | 108.8333 | 30.4000 |
|  | **Yunnan**, Weishan, Weibaoshan, 2200 m | 100.3000 | 25.2333 |
|  | Yiliang | 103.1500 | 24.9000 |
| *G. rhamphella* Wang & Sung* | **Anhui**, Yuexi | 116.3667 | 30.8667 |
|  | **Sichuan**, Emeishan | 103.3117 | 29.5333 |
| *G. rosanna* (Bleszynski)* | **Anhui**, Huangshan | 118.3000 | 29.7167 |
|  | Huoshan | 116.3167 | 31.4000 |
|  | **Fujian**, Wuyishan | 116.7000 | 26.9000 |
|  | **Guangdong**, Dadongshan, 650 m | 115.3833 | 23.3333 |
|  | **Guangxi**, Rongshui, 650 m | 109.2167 | 25.0667 |
|  | **Guizhou**, Chishui, 390 m | 105.7000 | 28.5667 |
|  | Xishui, 550 m | 106.2000 | 28.3167 |
|  | **Henan**, Neixiang, 650 m | 111.0833 | 33.0333 |
|  | **Hong Kong**, New Territories, 335 m | 114.1667 | 22.4167 |
|  | **Hubei**, Hefeng, 1260 m | 110.0333 | 29.8833 |
|  | **Hunan**, Zhangjiajie | 110.4333 | 29.8167 |
|  | **Zhejiang**, Tianmushan | 119.5667 | 30.4333 |
| *G. rosannoides* (Bleszynski)* | **Hubei**, Shennongjia | 116. 6667 | 30.2000 |
|  | **Sichuan**, Emeishan | 103.3117 | 29.5333 |
|  | **Zhejiang**, Tianmushan | 119.5667 | 30.4333 |
| *G. scrotiformis* Li & Li* | **Tibet**, Mêdog | 95.3000 | 29.2167 |
| *G. setacea* Song & Chen* | **Guizhou**, Daozhen, 1300–1370 m | 107.6000 | 28.8833 |
|  | Jiangkou, 1700 m | 108.0833 | 27.6833 |
|  | **Hubei**, Wufeng | 116.6667 | 30.2000 |
|  | Xianfeng, 1280 m | 109.1333 | 29.6667 |
| *G. siciformis* Li & Li* | **Fujian**, Tianzhushan | 118.0894 | 24.4796 |
|  | **Guangxi**, Longsheng | 110.0333 | 25.7833 |
|  | **Hainan**, Bawangling | 109.0500 | 19.2667 |
|  | Jianfengling, 810 m | 108.7833 | 18.7000 |
|  | Wanning | 110.4000 | 18.8000 |
|  | Wuzhishan | 109.6833 | 18.8833 |
|  | **Hong** **Kong**, New Territories, 200–315 m | 114.1667 | 22.4167 |
|  | **Yunnan**, Mengla | 101.5500 | 21.4833 |
|  | Puer | 100.5800 | 22.4800 |
| *G. sperlingi* Li, sp. nov.* | **Sichuan**, Mabian, 1100 m | 103.5167 | 28.8500 |
| *G. spiculella* Wang & Sung* | **Tibet**, Mêdog | 95.3000 | 29.2167 |
| *G. spinulella* Wang & Sung* | **Jiangxi**, Lushan | 115.9667 | 29.5000 |
|  | **Tibet**, Bomi | 95.7500 | 29.8833 |
| *G. subalbilinealis* (Bleszynski)* | **Anhui**, Jiuhuashan | 117.8000 | 30.3833 |
|  | Yuexi, Wenquan | 116.3667 | 30.8667 |
|  | **Fujian**, Nanping, 850 m | 118.1667 | 26.6333 |
|  | Wuyishan, 1100 m | 116.7000 | 26.9000 |
|  | **Guangdong**, Xinyi, Dawuling, 1000 m | 110.9333 | 22.3500 |
|  | **Guangxi**, Fangchenggang | 108.3333 | 21.6167 |
|  | Jinxiu, 550–800 m | 110.1833 | 24.1333 |
|  | Maoershan | 110.4167 | 25.8833 |
|  | Rongshui, 579 m | 109.2167 | 25.0667 |
|  | Shangsi, 250–510 m | 107.9667 | 22.1500 |
|  | **Guizhou**, Daozhen, 600 m | 107.6000 | 28.8833 |
|  | Fanjingshan, 530/2100 m | 108.6833 | 27.9167 |
|  | **Henan**, Tongbai, 300 m | 113.4000 | 32.3500 |
|  | **Hong Kong**, New Territories, 210–700 m | 114.1667 | 22.4167 |
|  | **Hubei**, Hefeng, 1260 m | 110.0333 | 29.8833 |
|  | Wufeng, 1000 m | 116.6667 | 30.2000 |
|  | Xianfeng, 1280 m | 109.1333 | 29.6667 |
|  | **Hunan**, Zhangjiajie, 650 m | 110.4333 | 29.8167 |
|  | **Shaanxi**, Huoditang, 1620 m | 108.4333 | 33.4333 |
|  | Taibaishan, 1700 m | 107.7833 | 33.9500 |
|  | **Sichuan**, Baoxing, 1100–1600 m | 102.8333 | 30.3667 |
|  | Emeishan | 103.3117 | 29.5333 |
|  | Mabian, 1500 m | 103.5167 | 28.8500 |
|  | Qingchengshan | 103.5167 | 30.9667 |
|  | Tianquan, 1300 m | 102.7667 | 30.0100 |
|  | Wolong, 2008 m | 103.6000 | 31.4833 |
|  | **Yunan**, Lijiang, 2650 m | 100.2333 | 26.8667 |
|  | **Zhejiang**, Lin’an, 420 m | 118.9333 | 30.0667 |
| *G. taeniata* Wang & Sung* | **Chongqing** | 106.5500 | 29.5833 |
|  | **Fujian**, Longqishan | 117.4667 | 26.7333 |
| *G. taphrophracta* (Meyrick)* | **Hubei**, Hefeng, 1260 m | 110.0333 | 29.8833 |
|  | Wufeng, 1000–1100 mm | 116.6667 | 30.2000 |
|  | **Guangxi**, Maoershan | 110.4167 | 25.8833 |
|  | **Guizhou**, Fanjingshan, 2100 m | 108.6833 | 27.9167 |
|  | **Sichuan**, Emeishan | 103.3117 | 29.5333 |
|  | Wolong, 2008 m | 103.6000 | 31.4833 |
| *G. tibetensis* (Wang & Sung)* | **Tibet**, Cuonale | 91.9333 | 27.9667 |
| *G. tridentata* Li & Li* | **Guizhou**, Daozhen, 600–1350 m | 107.6000 | 28.8833 |
|  | Xishui, 500 m | 106.2000 | 28.3167 |
|  | **Hubei**, Wufeng, 1000 m | 116.6667 | 30.2000 |
|  | **Yunan**, Kunming, 1900 m | 102.4500 | 24.9667 |
|  | **Zhejiang**, Tianmushan, 500 m | 119.5667 | 30.4333 |
| *G. tripunctata* (Moore) | **Fujian**, Wuyishan, 740–1100 m | 116.7000 | 26.9000 |
|  | **Guangxi**, Longsheng | 110.0333 | 25.7833 |
|  | Maoershan | 110.4167 | 25.8833 |
|  | **Zhejiang**, Taishun, 680–790 m | 119.7000 | 27.5500 |
| *G. vermeeri* (Bleszynski) | **Jilin**, Changbaishan | 125.6500 | 43.3500 |

Remarks: The asterisk (*) showing the species have been described from China as type locality; the pound (#) showing the detailed site of the species is unknown; *G. setosa* Li & Li was ignored in the analysis of *Glaucocharis* distribution, which could be transferred to Cybalomiinae.

**Table S2.** Environmental variables used in the study and their percentage contribution and permutation importance

| Code | Environmental variables | Unit | Percent Contribution | Permutation importance |
| --- | --- | --- | --- | --- |
| Bio1 | Annual mean temperature | ◦C | 0.4 | 1.5 |
| Bio2 | Mean diurnal range (mean of monthly max. and min. temp.) | ◦C | **15.8** | 6.5 |
| Bio3 | Isothermality ((Bio2/Bio7) × 100) | – | 0.4 | 1.1 |
| Bio4 | Temperature seasonality (standard deviation ×100) | C of V | 1.1 | 0.5 |
| Bio5 | Maximum temperature of warmest month | ◦C | 0.7 | 0.9 |
| Bio6 | Minimum temperature of coldest month | ◦C | **13** | 1.5 |
| Bio7 | Temperature annual range (Bio5–Bio6) | ◦C | 1.1 | 1.9 |
| Bio8 | Mean temperature of wettest quarter | ◦C | 0.6 | 1.6 |
| Bio9 | Mean temperature of driest quarter | ◦C | 0.3 | 3.2 |
| Bio10 | Mean temperature of warmest quarter | ◦C | 1.1 | 12.6 |
| Bio11 | Mean temperature of coldest quarter | ◦C | 6.7 | 4.7 |
| Bio12 | Annual precipitation | mm | 0.3 | 4 |
| Bio13 | Precipitation of wettest period | mm | 3 | 5 |
| Bio14 | Precipitation of driest period | mm | 10.4 | 4.5 |
| Bio15 | Precipitation seasonality (CV) | C of V | 4.4 | 0.5 |
| Bio16 | Precipitation of wettest quarter | mm | 0.2 | 0.7 |
| Bio17 | Precipitation of driest quarter | mm | 1.3 | 1.5 |
| Bio18 | Precipitation of warmest quarter | mm | **35** | 37.7 |
| Bio19 | Precipitation of coldest quarter | mm | 0.6 | 2 |
| Alt | Altitude above sea level | m | 3.6 | 8.2 |
